# The Active Constituent From Gynostemma Pentaphyllum Prevents Liver Fibrosis Through Regulation of the TGF-β1/NDRG2/MAPK Axis

**DOI:** 10.3389/fgene.2020.594824

**Published:** 2020-11-04

**Authors:** Hui Huang, Kuifeng Wang, Qian Liu, Feihong Ji, Hu Zhou, Shanhua Fang, Jiansheng Zhu

**Affiliations:** ^1^Department of Analytical Chemistry and CAS Key Laboratory of Receptor Research, Shanghai Institute of Materia Medica, Chinese Academy of Sciences, Shanghai, China; ^2^University of Chinese Academy of Sciences, Beijing, China; ^3^Department of Infectious Diseases, Affiliated Taizhou Hospital of Wenzhou Medical University, Taizhou, China; ^4^Suzhou GenHouse Pharmaceutical Co., Ltd., Suzhou, China

**Keywords:** HSC, TGF-β1, liver fibrosis, quantitative proteomics, NDRG2

## Abstract

Liver fibrosis resulting from chronic liver damage constitutes a major health care burden worldwide; however, no antifibrogenic agents are currently available. Our previous study reported that the small molecule NPLC0393 extracted from the herb *Gynostemma pentaphyllum* exerts efficient antifibrotic effects both *in vivo* and *in vitro*. In this study, a TMT-based quantitative proteomic study using a carbon tetrachloride (CCl_4_)-induced mouse model of liver fibrosis was performed to identify the potential target of NPLC0393. Combining this study with bioinformatic analysis of differentially expressed proteins between the CCl_4_ model and NPLC0393 treatment groups, we focused on the function of N-myc downstream-regulated gene 2 (NDRG2) involved in cell differentiation. *In vitro* studies showed that NPLC0393 prevented the TGF-β1 stimulation-induced decrease in the NDRG2 level in hepatic stellate cells (HSCs). Functional studies indicated that NDRG2 can inhibit the activation of HSCs by preventing the phosphorylation of ERK and JNK. Furthermore, knockdown of NDRG2 abolished the ability of NPLC0393 to inhibit HSC activation. In conclusion, these results provide information on the mechanism underlying the antifibrotic effect of NPLC0393 and shed new light on the potential therapeutic function of the TGF-β1/NDRG2/MAPK signaling axis in liver fibrosis.

## Introduction

Liver fibrosis is a reversible wound healing response that occurs in chronic liver injury and is caused by viral infection, drug exposure, and metabolic and autoimmune disorders (Parola and Pinzani, 2019). Liver fibrosis is characterized by deposition of scar tissue, i.e., extracellular matrix (ECM), which may further lead to cirrhosis accompanied by portal hypertension, liver failure, and/or liver cancer. Activation of hepatic satellite cells (HSCs) is a crucial event in fibrogenesis (Tsuchida and Friedman, 2017). In the context of liver injury, HSCs undergo transdifferentiation from quiescent cells to proliferative, contractile, and fibrogenic myofibroblasts that are capable of excessive production and secretion of ECM. The progression and resolution of fibrosis are complex processes, involving interactions among cells, soluble mediators (e.g., Tumor necrosis factor alpha, TNF-α), the ECM and intracellular signaling events (e.g., TGF-β1/Smad signaling) relevant to the fibrogenic process (Hernandez-Gea and Friedman, 2011). Given that fibrosis is the primary predictor of liver-related morbidity and mortality, the development and testing of antifibrotic strategies that can prevent, halt, or even reverse liver fibrosis are urgently needed (Bottcher and Pinzani, 2017).

The natural compound NPLC0393 is a triterpene saponin constituent isolated from the Chinese herb *Gynostemma pentaphyllum* (Yin and Hu, 2005). *Gynostemma pentaphyllum* is a popular herbal medicine in Asia, and it was proven safe in a chronic toxicology study (Attawish et al., 2004). A previous study suggested that NPLC0393 could function as a small molecule activator of Serine/Threonine Phosphatase PP2Cα and exert significant antifibrotic effects in rat models of carbon tetrachloride (CCl_4_) - or bile duct ligation (BDL)-induced fibrosis (Wang et al., 2010). Mechanistic experiments suggested that NPLC0393 inhibits HSC activation by blocking both the canonical TGF-β1/Smad signaling pathway and the TGF-β1/p65/MAT2A axis (Wang et al., 2010, 2019). However, the possible involvement of other mechanisms underlying the antifibrotic effect of NPLC0393 should not be ruled out, since multiple effectors are likely critical for the full fibrotic response to TGF-β1 signaling (Walton et al., 2017). Therefore, it is meaningful to comprehensively explore the molecular mechanism underlying the TGF-β1-mediated activation of HSC.

N-myc downstream-regulated gene 2 (NDRG2) is a member of the NDRG family that is involved in cell proliferation and differentiation. Under normal physiological conditions, NDRG2 plays an important role in liver histogenesis and maintenance, as the NDRG2 expression level is dynamically increased during embryonic liver development, and NDRG2 is modestly expressed in normal liver tissue (Hu et al., 2006). However, loss of NDRG2 expression frequently occurs at both the mRNA and protein levels in carcinoma (Lee et al., 2008). Therefore, NDRG2 has recently been recognized as a tumor suppressor in various cancers. In addition, some researchers have demonstrated the antifibrotic role of NDRG2 based on experimental data (Yang et al., 2011; Jin et al., 2017). However, it’s exact mechanism of antifibrosis has not been elucidated.

In this study, we found via a tandem mass tag (TMT)-based quantitative proteomic approach that NDRG2 was significantly differentially expressed between the normal, CCl_4_-induced fibrosis model and NPLC0393-treated groups of mice. Our *in vitro* study revealed that NDRG2 functions as an inhibitor of the mitogen-activated protein kinase (MAPK, i.e., ERK1/2 and JNK) signaling pathway and that NPLC0393 inhibits HSC activation induced by TGF-β1 by upregulating NDRG2 expression. Our results may provide new insight into the non-canonical TGF-β1 signaling targeted therapies for liver fibrosis.

## Materials and Methods

### Animal Experiment

**FIGURE 1:**
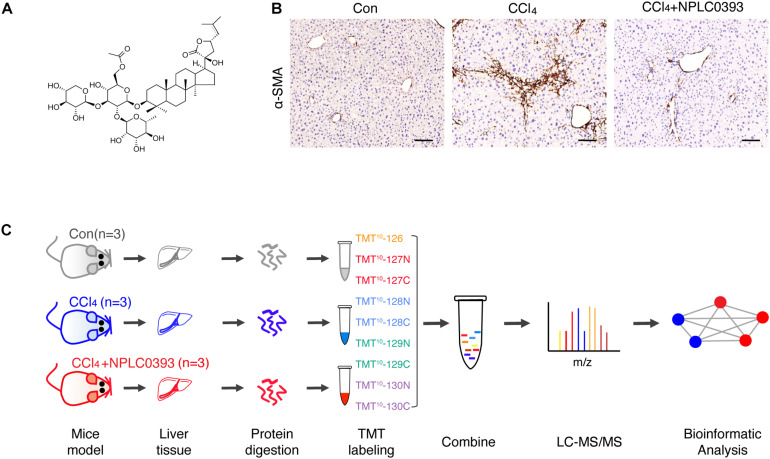
Quantitative proteomic analysis of mice liver tissue from different treatment groups. **(A)** Chemical structure of NPLC0393. **(B)** Liver sections from mice in the control (Con), CCl_4_ model (CCl_4_), and CCl_4_ model combined with NPLC0393 treatment (CCl_4_ + NPLC0393) groups were immunohistochemically stained with an anti-α-SMA antibody (scale bar, 100 μm). **(C)** Schematic of the experimental setup.

In the animal experiment, male C57BL/6 mice (6 weeks, weighing 18–20 g, provided by Shanghai SLAC Laboratory Animal Co., Ltd.) were divided into three groups (*n* = 7 per group): the normal group (Con), in which the mice received daily intraperitoneal doses of olive oil; the CCl_4_ model group (CCl_4_), in which the mice received intraperitoneal doses of CCl_4_ (diluted 1:10 in olive oil, 0.5 mL/kg, twice weekly for 4 weeks); and the NPLC0393 treatment group (CCl_4_ + NPLC0393), in which the mice received intraperitoneal doses of NPLC0393 (dissolved in PBS; 5 mg/kg, daily) combined with CCl_4_ injection for 4 weeks. NPLC0393 was provided by the laboratory of Prof. Lihong Hu, National Center of Drug Screening, Shanghai Institute of Materia Medica (Yin and Hu, 2005). The structure of NPLC0393 is shown in Figure 1A. All mice were housed under standard conditions and were sacrificed at the experimental endpoint. Liver samples were fixed with 10% buffered formalin or snap frozen in liquid nitrogen and stored at −80°C. Three mice from each group were used for the quantitative proteomic study, and the remaining four mice were used for validation by western blot analysis or immunohistochemistry. The animal experiment was approved by the Science and Technology Commission of Shanghai Municipality, and all experimental procedures were performed according to the ethical guidelines of the Animal Care and Use Committee, Shanghai Institute of Materia Medica, Chinese Academy of Sciences.

### Protein Extraction and Digestion

Liver tissues from a total of nine mice among all three groups were lysed with SDT lysis buffer [4% SDS, 0.1 M Tris-HCl (pH 7.6), 0.1 M DTT], and the protein concentration was measured by the tryptophan-based fluorescence quantification method (Thakur et al., 2011). The sample quality was determined by SDS–PAGE and silver staining before conducting subsequent experiment (Supplementary Figure A). For protein digestion, 50 μg of each sample was processed with the filter-assisted sample preparation (FASP) method, as previously described (Wisniewski et al., 2010). In brief, protein samples were loaded in 30-kDa centrifugal filter tubes (Millipore) and washed with 100 μL of UA buffer [8 M urea in 0.1 M Tris-HCl (pH 8.5)]. Alkylation of proteins was performed using 50 mM iodoacetamide (IAA, dissolved in UA buffer) in the dark at room temperature for 30 min. Proteins were washed with 100 μL of UA buffer and washed again with 100 μL of 100 mM triethyl ammonium bicarbonate (TEAB). Proteins were digested with trypsin (Promega) at a ratio of 1:50 (w/w) in 50 mM TEAB) overnight at 37°C. Peptides were dried by vacuum centrifugation for the subsequent TMT labeling experiment.

### TMT 10-plex Labeling

The peptides obtained from each sample were then labeled with one 10-plex TMT reagent set according to the instructions (Thermo Fisher Scientific, United States). Each TMT reagent (0.4 mg) was dissolved in 41 μL of anhydrous acetonitrile (ACN) and added to the corresponding peptide samples (resolved in 200 mL of 100 mM TEAB). Control samples were labeled with 126, 127N, and 127C; CCl_4_ group samples were labeled with 128N, 128C, and 129N; and CCl_4_ + NPLC0393 group samples were labeled with 130N, 130C, and 131N. After incubation for 1 h at room temperature, 8 μL of 5% hydroxylamine was added for 15 min to quench the labeling reaction. The labeled peptides were combined into a single sample, dried by vacuum centrifugation and subjected to solid-phase extraction and desalting on a C18 column (3M Empore) (Werner et al., 2014).

### High-pH Reverse Phase Liquid Chromatography (RPLC) Fractionation

High-pH RPLC was performed in an Agilent 1100 LC instrument equipped with a C18 column (Waters XBridge BEH300, 250 × 4.6 mm, OD 5 μm, United States) at a flow rate of 0.7 mL/min. The pooled peptides were fractionated into 24 fractions using a gradient of mobile phases A (10 mM NH_4_COOH, pH 10) and B (90% acetonitrile in 10 mM NH4COOH, pH 10). Forty-eight fractions were collected over the entire gradient and combined into 24 samples with similar peptide amounts. The fractions were dried in a vacuum centrifuge.

### Nano-LC-MS/MS

The fractionated peptides (approximately 1 μg of each fraction) were resolved in 0.1% formic acid. MS analysis was performed in an Orbitrap Q-Exactive HF mass spectrometer coupled online to an EASY-nLC1000 HPLC system equipped with a nanoelectrospray ion source (all from Thermo Fisher Scientific, Germany). The peptides were loaded on a custom column (75 μm × 200 mm; 3 μm ReproSil-Pur C18 beads, Dr. Maisch GmbH, Germany) and eluted over 70 min with an LC gradient at a flow rate of 300 nL/min. Mobile phases A and B were 0.1% (v/v) formic acid in H_2_O and 0.1% (v/v) formic acid in acetonitrile, respectively. The mobile phase gradient conditions were as follows: 2–5% B for 1 min; 5–27% B for 53 min; 27–40% B for 10 min; 40–90% B for 2 min; 90% B for 4 min. The electrospray voltage was 2.5 kV in positive ion mode, and the capillary temperature was 275°C. Data were acquired in data-dependent mode using Xcalibur software. MS1 spectra were acquired in the Orbitrap analyzer with a resolution of 120,000 @ m/z 200, an automatic gain control (AGC) target of 3 × 10^6^, a maximum ion time (IT) of 50 ms and a scan range of 350–1,700 m/z. This scan was followed by 15 targeted MS2 scans acquired by high-energy collision dissociation (HCD) with the following parameters: a resolution of 60,000 @ m/z 200, an AGC target of 1 × 10^5^, a maximum IT of 120 ms, an isolation window of 1 m/z, a normalized collision energy (NCE) of 32%, a fixed first mass of 105 m/z, and a profile spectrum data type. Precursors with charge states of 1, 7, 8, and >8 were excluded from MS2 analysis.

For accurate mass measurements, the lock mass option was employed. Dynamic exclusion was set at 30 s. Application of the mass spectrometer scan functions and HPLC solvent gradients was controlled by the Xcalibur data system (Thermo Scientific).

### Protein Identification, Quantification

Raw MS files were analyzed using MaxQuant software (version 1.6.5.0^1^ (Cox et al., 2009). MS/MS spectra were searched with the Andromeda search engine against the UniProt Mus musculus database downloaded from Uniprot (accessed June 2020^2^). TMT 10-plex-based MS2 reporter ion quantification was selected, with the reporter mass tolerance set at 0.003 Da. Methionine oxidation, protein N-terminal acetylation and lysine acetylation were set as the variable modifications. Trypsin/P was selected as the digestive enzyme, with two potential missed cleavages. The false discovery rate (FDR) was set to 1% for peptide and protein identification. The labeling efficiency was estimated to be 94% by calculating the percentage of modified N-terms and lysines. The mass spectrometry proteomic data have been deposited to the ProteomeXchange Consortium via the PRIDE partner repository with a dataset identifier (project accession no. PXD019448).

### Proteomic Data Analysis

The raw data of the protein table were filtered to eliminate identifications from the reverse database and common contaminants. Each confident protein assignment was required to contain at least 1 unique peptide. Data were normalized using the median centering method to correct sample loading differences and were log2-transformed. For comparison of three groups, one-way analysis of variance (ANOVA) followed by Benjamini and Hochberg was performed with R language (4.0.2). Tukey’s honest significant difference (HSD) test was performed for comparing two groups. Fold change > 1.5 and *P* < 0.01 were set as the criteria for significant changes between any of the two groups.

### Bioinformatic Analysis

**FIGURE 2:**
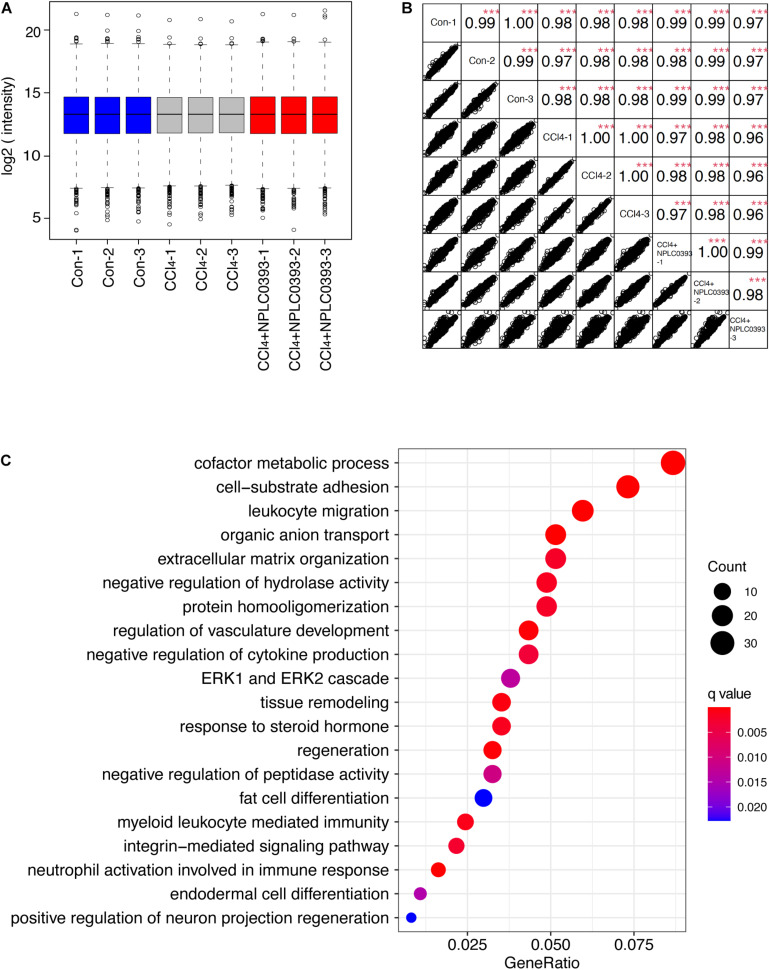
Identification of potential target of NPLC0393 by bioinformatic analysis. **(A)** Boxplots analysis of protein intensity across all samples. **(B)** Correlation analysis between each two samples. Rows and columns represent samples, and each square shows the correlation coefficients (Spearman *r*) between two samples. ***P < 0.001 comparing intensity of each two samples. **(C)** GOBP enrichment analysis of the differentially expressed proteins. Values are expressed as GeneRatio and color scale bar indicates adjusted *P*-value. Node size is proportional to the number of genes associated with the GO category.

Boxplots in the Figure 2A was generated using the boxplot function of R language. The similarity of proteins between each two biological replicates was assessed using the Spearman’s rank correlation coefficient. Hierarchical clustering of proteins was performed using Euclidean distances in the ‘pheatmap’ package (1.0.12) (Hochfellner et al., 2014). PCA analysis was done using the FactoMineR package (1.3.4) (Jombart, 2008). Gene Ontology (GO) functional enrichment analysis of biological processes was performed using R ClusterProfiler package (3.16.1) and original GO terms were simplified using “simplify” function of ClusterProfiler (Yu et al., 2012). To identify the significant terms *q* values were estimated using the Benjamini–Hochberg approach, and *q* < 0.05 were considered to be significantly enriched. All of these analyses were performed in the R language 4.0.2.

### Histological Analysis

Liver tissues were immediately formalin fixed, paraffin embedded and sectioned. Immunohistochemistry with an anti-α-SMA antibody (diluted 1:100 with PBS, Abcam, Cambridge, United Kingdom) was performed on the samples. The stained tissue samples were examined using an Olympus BX41 microscope (Olympus Corporation, Japan). Image-Pro^®^ plus 4.5 image analysis software (Media Cybernetics, United States) was used to evaluate qualitative and quantitative changes.

### Cell Culture and Treatment

The human HSC cell line LX-2 (provided by the Institute of Liver Diseases, Shanghai University of Traditional Chinese Medicine) was routinely cultured in Dulbecco’s minimum essential medium (DMEM) containing 10% fetal bovine serum (FBS) in an atmosphere of 5% CO_2_ at 37°C. Cells were treated with increasing concentrations (1, 5, and 10 μM) of NPLC0393 (dissolved in DMSO) for 24 h and further stimulated with 10 ng/mL TGF-β1 (R&D Systems) for another 24 h. DMSO was used as a positive control.

Specific siRNAs targeting the NDRG2 gene (siNDRG2; sequence: 5′-CUGGCGAGAUAUGCUCUUATT-3′) and a negative control non-targeting siRNA (siNC; sense, sequence: 5′-UUCUCCGAACGUGUCACGUTT-3′) were synthesized by GenePharma (Shanghai, China). At a confluence of approximately 70%, LX-2 cells were transfected separately with these oligonucleotides at a final concentration of 100 nM using Lipofectamine 3000 (Invitrogen, United States). After 24 h of transfection, HSCs were treated with NPLC0393 for 24 h, further treated with the ERK inhibitor U0126 (Sigma, United States) or the JNK inhibitor SP600125 (Sigma, United States) for 2 h, and finally stimulated with TGF-β1 for another 24 h. The experiment was repeated at least three times.

### Quantitative Real-Time PCR (qRT-PCR)

Total RNA was extracted from cultured LX-2 cells using TRIzol reagent (Invitrogen, United States). First-strand complementary cDNA was synthesized with random hexamer primers and SuperScript III reverse transcriptase (Life Technologies, United States). Quantitative PCR was performed in a 7500 Fast Real-Time PCR system (Applied Biosystems, Irvine, CA, United States) using real-time fluorescent quantitative reagents (TAKARA).

The following primers were used in this study: NDRG2 (5′-ATGCTCTTAACCACCCGGACA-3′, 5′-ACGCTCAAAGT TCAGGTCTCG-3′), ACTA2 (5′-CGTGGCTATTCCTTCGTT AC-3′, 5′-TGCCAGCAGACTCCATCC-3′), COL1A1 (5′-CC GTGACCTCAAGATGTGCCACT-3′, 5′-TCATGCTCTCGCCG AACCAG-3′), and GAPDH (5′-GGTATCGTGGAAGGA CTCATGAC-3′, 5′-ATGCCAGTGAGCTTCCCGTTCAGC-3′). These primers were purchased from Shanghai Generay Biotech Co., Ltd. (Shanghai, China).

### Western Blot Analysis

Liver tissue or LX-2 cells were lysed with 4% SDS lysis buffer containing 0.1 M DTT. Protein lysates were separated by 10% SDS-PAGE and transferred to polyvinylidene fluoride (PVDF) membranes, which were then blocked with 5% fat-free milk at room temperature for 1 h and incubated with anti-NDRG2 (Abcam, United Kingdom), anti-phospho-ERK (Santa Cruz, United States), anti-ERK (Santa Cruz, United States), anti-phospho-JNK (Santa Cruz, United States), anti-JNK (Santa Cruz, United States), anti-α-SMA (Abcam, United Kingdom), anti-COL1A1 (Novus, United States), and anti-GAPDH (Sigma, United States) antibodies at 4°C overnight. After three washes in TBST, membranes were incubated with a goat anti-rabbit secondary antibody (Invitrogen, United States) or a goat anti-mouse secondary antibody (Invitrogen, United States) for 1 h at room temperature. Super Signal West Pico Chemiluminescent Substrate (Thermo Scientific, United States) was used to visualize antigens. Blots were scanned using ImageQuant (GE Healthcare, United Kingdom), and the resulting images were quantitatively analyzed in ImageJ software. The experiment was repeated at least three times.

### Statistical Analysis

The biological experiment data are presented as the mean ± SD values. Statistical analysis was performed using GraphPad Prism 6.0 (GraphPad Software, CA). Assumptions of normality were assessed using Shapiro–Wilk normality tests. One-way ANOVA followed by Dunnett’s *post hoc* test was used for multiple comparison analyses. Two-sided *P*-values of <0.05 were considered statistically significant.

## Results

### Identification of the Differentially Expressed Protein NDRG2 by TMT-Based Quantitative Proteomic Analysis

In this study, we first confirmed using a CCl_4_-induced mouse model that NPLC0393 efficiently alleviates liver fibrosis (Figure 1B). To discover proteins associated with the protective effects of NPLC0393 against CCl_4_-induced liver fibrosis and identify potent therapeutic targets, we performed TMT-LC-MS/MS-based proteomic studies of liver tissues obtained from normal mice (Con), mice with CCl_4_-induced fibrosis (CCl_4_) and mice with CCl_4_-induced fibrosis treated with NPLC0393 (CCl_4_ + NPLC0393) (*n* = 3 per group). The workflow of the proteomic study is shown in Figure 1C.

With criteria of an FDR of <1% for both peptide and protein identification, we quantified a total of 7344 proteins across 9 samples (Supplementary Tables S1, S2). A boxplot analysis revealed similar average reporter ion intensities across 9 samples (Figure 2A), indicating that TMT experimental procedures showed no bias toward different samples.

Furtherly, the correlation coefficient of the log2 TMT ion intensities between any two cohorts was greater than 0.96 (Figure 2B), demonstrating the consistent stability of the MS platform. The principal component analysis (PCA) of 7344 proteins showed that 9 samples can be separated into three independent clusters according to different treatment (Supplementary Figure B). Hierarchical clustering of these proteins also successfully classified the liver tissue samples into the normal, model and drug-treated groups, suggesting that the different treatments profoundly affected the protein expression level (Supplementary Figure C).

Then, we screened significantly changed proteins between each pair of groups (Con vs. CCl_4_ and CCl_4_ vs. CCl_4_ + NPLC0393) using a criterion of *P*-value < 0.01 and fold change > 1.5. Compared to normal mice, CCl_4_ mice exhibited significant alterations in 715 proteins. Of these, 241 and 172 proteins were upregulated and downregulated, respectively, as compared to control group, while they were remarkably reversed to basal levels in the CCl_4_ + NPLC0393 group, which was similar to that of control group. We considered these 413 proteins as biological meaningful proteins which may be related to the anti-fibrotic effect of NPLC0393 (Supplementary Table S3).

To identify the potential effector for NPLC0393 in regulating liver fibrogenesis, we performed Gene Ontology (GO) enrichment analysis for the differentially expressed proteins. GO biological process (GOBP) annotations revealed that NPLC0393 treatment modulated high percentage of proteins that regulate the metabolism, such as cofactor metabolic process, organic anion transport, regulation of hydrolase activity and protein homooligomerization. On the other hand, the results also reveal the over-representation of proteins associated with cell adhesion, regulation of cytokine production, regulation of vasculature development, as well as ECM organization (Figure 2C and Supplementary Table S4). Interestingly, the ERK cascade were ranked top ten in the GOBP terms. It was well known that TGF-β1, a highly potent fibrogenic cytokine can activate both Smad and mitogen-activated protein kinase (MAPK) signaling pathways, including ERK, p38 and JNK, to promote HSC activation (Tsuchida and Friedman, 2017). Among the proteins involved in ERK cascade (Table 1), NDRG2 is of biological interest since it also can regulate cell differentiation and cytokine production (Shen et al., 2014, 2018). As HSC become activated through a process of trans-differentiation into Myofibroblast-like cells, we suspected that NDRG2 may mediate the TGF-β1-induced HSC activation via regulating MAPK signaling. Taken together, based on the bioinformatic analysis and literature review, we sought to determine the potential function of NDRG2 in HSC activation.

**TABLE 1:** List of genes enriched in the Top 10 GO terms.

**GO ID and description**	**Generatio**	**qvalue**	**Gene ID**
GO:0051186 cofactor metabolic process	0.086	6.12E-11	Acnat1/Prdx6/Cyp1a2/Alas2/Alad/Gpx1/Dnmt1/Hmox1/Gstp1/Cat/Gsta3/Akr1c6/Gcdh/Gstt2/Rgn/Acsm5/Acnat2/Ido2/Flad1/Ces1d/Acsm1/Mgst1/Far1/Mpv17l/Nudt7/Glo1/Coasy/Gstk1/Hpgds/Nat8/Pdk2/Gnmt
GO:0031589 cell-substrate adhesion	0.073	7.76E-09	Itga8/Myadm/Itgb5/Coro1a/Lamb1/Spp1/Fn1/Itgb2/Plg/Vcam1/Vtn/Mmp12/Fbln2/Alox15/Cd63/Itgav/Mmp14/Sirpa/Map4k4/Fbln1/Fndc3b/Flna/Fermt3/Emilin1/Parvg/Iqgap1/Enpp2
GO:0050900 leukocyte migration	0.060	3.09E-06	Stk10/Coro1a/Itgam/Anxa1/Spp1/Itgb2/App/Lgals3/Plg/Il1rn/Vcam1/Mif/Mmp14/Sirpa/Abr/Ano6/Nckap1l/Emilin1/Trem2/Rtn4/Cd200r1/Stk39
GO:0015711 organic anion transport	0.051	7.32E-06	Agxt/Slc1a4/Anxa1/Slc3a2/Il1rn/Mif/G6pc/Slc1a2/Ano6/Slc25a24/Slc25a1/Slc25a42/Tmem30a/Pla2g12a/Slco1b2/Slc7a8/Slc25a13/Slco1a1/Slc37a2
GO:0030198 extracellular matrix organization	0.051	9.08E-06	Sh3pxd2b/Itga8/Ctss/Lamb1/Anxa2/Fn1/App/Lgals3/Serpinh1/Plg/Vtn/Mmp12/Fbln2/Mmp14/Fbln1/Aebp1/Emilin1/Mmp19/Efemp2
GO:0051346 negative regulation of hydrolase activity	0.049	0.000174	Itih4/Anxa1/Gpx1/App/Serpinh1/Timp2/Vtn/Serpina7/Mcm2/Slpi/Serpina3m/Ambp/Hgf/Rgn/Ppp1r9b/Nckap1l/Amot/Naip2
GO:0051260 protein homooligomerization	0.049	0.000187	Hsd17b10/Slc22a1/Aldoc/Alad/App/Hmox1/Glul/Cat/Alox5ap/Mif/Slc1a2/Evl/Clu/Rhoc/Mgst1/Emilin1/Gnmt/Ak3
GO:1901342 regulation of vasculature development	0.043	0.000444	Anxa3/Anxa1/Itgb2/Dnmt1/Hmox1/Glul/Fgf2/Lgals3/Plg/Bmp7/S100a1/Hgf/Amot/Emilin1/Rtn4/Enpp2
GO:0001818 negative regulation of cytokine production	0.043	0.000807	Tyrobp/Anxa1/Fn1/Hmox1/Gstp1/Sirpa/Acp5/Hgf/Fbln1/Cd84/Nckap1l/Trem2/Gpnmb/Cd200r1/Prg4/**Ndrg2**
GO:0070371 ERK1 and ERK2 cascade	0.038	0.001141	Fn1/App/Fgf2/Gstp1/Mif/Alox15/Itgav/Sirpa/Fbln1/Emilin1/Trem2/Gpnmb/Nqo2/**Ndrg2**

*GO ID: Gene Ontology identifier; Generatio: the ratio of the genes in the associated GO term against the number of all input genes; Gene ID: Gene name of proteins that enriched in the GO terms; qvalue: Benjamini-Hochberg adjusted P value.*

### NPLC0393 Upregulates NDRG2 in the Fibrotic Liver and TGF-β1-Activated HSCs

**FIGURE 3:**
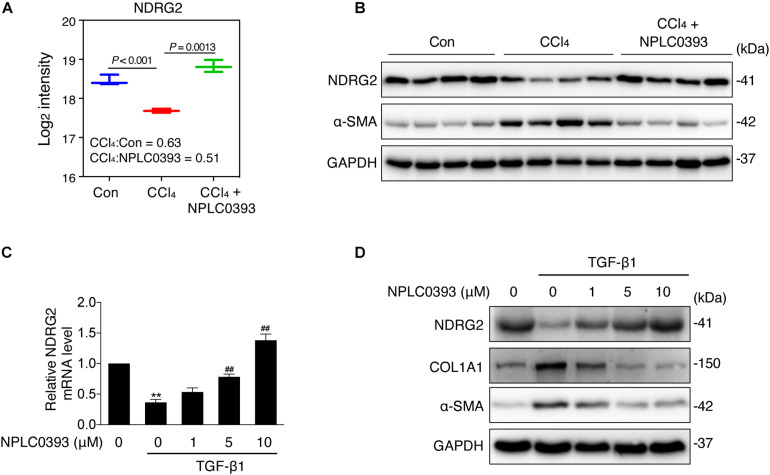
Effect of NPLC0393 on the expression level of NDRG2. **(A)** Boxplot showing the log2 transformed intensity of NDRG2, as quantified by LC-MS/MS. **(B)** Western blot of NDRG2 (∼41 KD) and α-SMA protein expression in all tissue samples (*n* = 4 per group). GAPDH was used as the loading control. **(C)** LX-2 cells were treated with increasing concentrations (1, 5, and 10 μM) of NPLC0393 for 24 h and stimulated with 10 ng/mL TGF-β1 for another 24 h. Relative mRNA levels of NDRG2 were analyzed by qRT-PCR. **P < 0.01 compared with the control group; ^##^*P* < 0.01 compared with the TGF-β1 treatment group, as analyzed by one-way ANOVA followed by Dunnett’s test. **(D)** Representative western blot of NDRG2, α-SMA and COL1A1 protein expression in LX-2 cells treated as in **(C)**.

As shown in Figure 3A, the MS data showed that NDRG2 was downregulated by CCl_4_ intoxication (*P* < 0.001, CCl_4_: Con ratio = 0.63) and that this change was reversed by NPLC0393 treatment (*P* = 0.0013, CCl_4_: CCl_4_+NPLC0393 ratio = 0.51). These results were further confirmed by western blotting of liver tissues from another set of independent samples from the control, CCl_4_ and CCl_4_ + NPLC0393 groups (*n* = 4 per group, Figure 3B).

Next, we evaluated the effect of NPLC0393 on the expression level of NDRG2 in the human HSC cell line LX-2. Given that TGF-β1 is a crucial factor in the progression of liver fibrosis and has been reported to inhibit NDRG2 expression in HSCs, we validated the effects of NPLC0393 on TGF-β1-induced downregulation of NDRG2. LX-2 cells were treated with 0, 1, 5, or 10 μM NPLC0393 for 24 h and stimulated with 10 ng/mL TGF-β1 for another 24 h. The qRT-PCR and western blot results showed that NPLC0393 treatment dose-dependently restored TGF-β1-induced downregulation of NDRG2 (Figures 3C,D). In addition, NPLC0393 effectively inhibited HSC activation, as evidenced by the decrease in COL1A1 and α-SMA (encoded by ACTA2) expression. These results suggested that the antifibrotic effect of NPLC0393 might be attributed to its ability to induce NDRG2 expression in activated HSCs.

### NDRG2 Inhibits HSC Activation by Regulating the TGF-β1/MAPK Signaling Pathway

**FIGURE 4:**
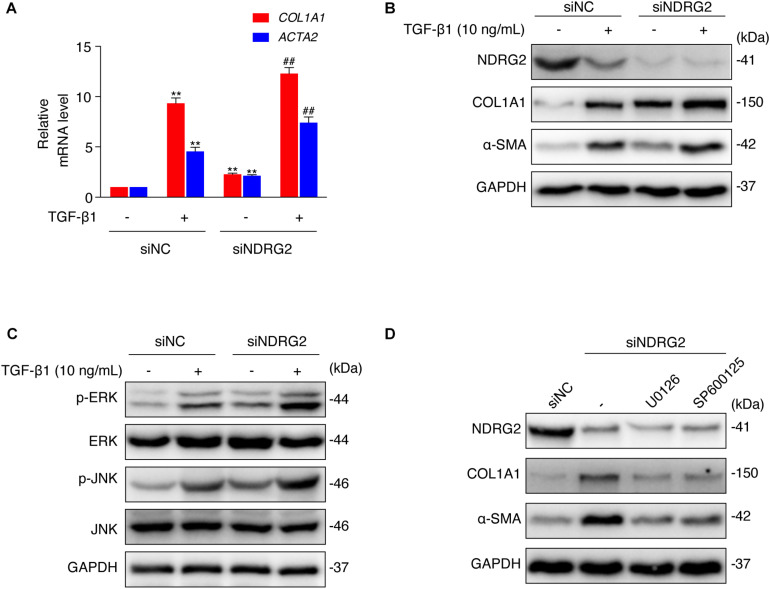
Effects of NDRG2 on HSC activation and TGF-β1/MAPK signaling pathway activity. **(A)** LX-2 cells were transfected with siNDRG2 or siNC for 24 h and stimulated with 10 ng/mL TGF-β1 for another 24 h. Relative mRNA levels of ACTA2 and COL1A1 were analyzed by qRT-PCR. **P < 0.01 compared with the control group; ^##^*P* < 0.01 compared with the TGF-β1 treatment group, as analyzed by one-way ANOVA followed by Dunnett’s test. **(B)** The protein levels of α-SMA and COL1A1 were analyzed by western blotting. **(C)** LX-2 cells were treated as described above, except that the cells were stimulated with TGF-β1 for 30 min, and the protein levels of total and phosphorylated JNK and ERK1/2 were analyzed by western blotting. **(D)** LX-2 cells were transfected with siNDRG2 and siNC for 24 h, treated with the ERK inhibitor U0126 (10 μM) or the JNK inhibitor SP600125 (50 μM) for 2 h. The protein levels of α-SMA and COL1A1 were analyzed by western blotting.

Then, we performed an *in vitro* loss-of-function study to evaluate the effect of NDRG2 on HSC activation. We transiently silenced NDRG2 expression in LX-2 cells using siRNA and stimulated cells with TGF-β1. As shown in Figures 4A,B, in NDRG2 knockdown LX-2 cells, both the mRNA and protein expression levels of α-SMA and COL1A1 were increased. TGF-β1 can promote HSC activation through a non-Smad pathway, which is mediated by MAPK family members such as ERK1/2, JNK and p38 MAPK (Zhang, 2009; Biernacka et al., 2011). Therefore, we examined the effect of NDRG2 on TGF-β1-induced activation of the MAPK pathway. The western blot results showed that NDRG2 deficiency increased both the basal and TGF-β1-stimulated phosphorylation levels of ERK1/2 and JNK (Figure 4C). To determine the role of MAPK activation in the antifibrotic effect of NDRG2, we silenced the NDRG2 gene in LX-2 cells and treated cells with the ERK inhibitor U0126 and the JNK inhibitor SP600125. Compared to siNC transfection, siNDRG2 transfection enhanced the expression levels of α-SMA and COL1A1; however, these changes were reversed by treatment with MAPK inhibitors (Figure 4D). These results suggested that NDRG2 can prevent HSC activation by regulating the TGF-β1-MAPK signaling pathway.

### The Inhibitory Effect of NPLC0393 on HSC Activation Requires NDRG2

**FIGURE 5:**
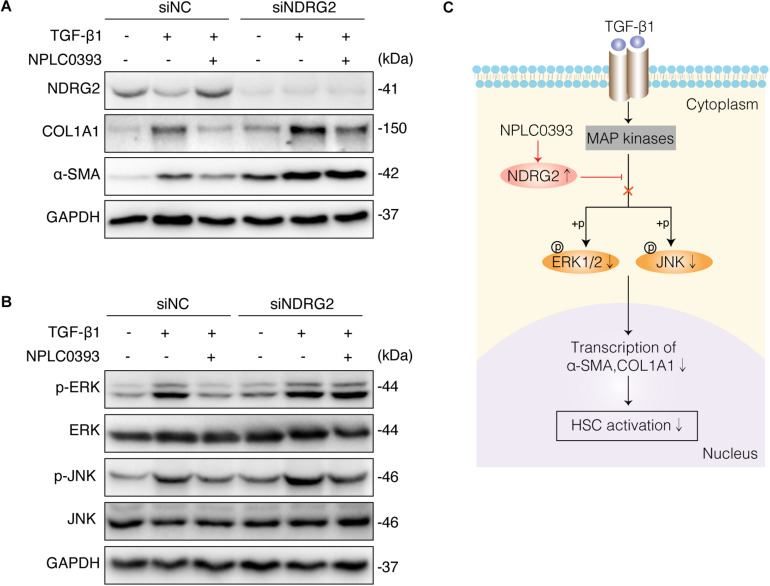
The role of NDRG2 in mediating the antifibrotic effects of NPLC0393. **(A)** LX-2 cells transfected with siNDRG2 or siNC for 24 h were treated with NPLC0393 for 24 h and further stimulated with TGF-β1 for another 24 h. The protein levels of α-SMA and COL1A1 were analyzed by western blotting. **(B)** LX-2 cells were treated as described above, except that the cells were stimulated with TGF-β1 for 30 min, and the protein levels of total and phosphorylated ERK1/2 and JNK were analyzed by western blotting. **(C)** Schematic showing the mechanism underlying the protective effect of NPLC0393 against hepatic fibrosis. NPLC0393 inhibits TGF-β1-induced downregulation of NDRG2. In turn, NDRG2 upregulation disrupts the transcription of α-SMA and COL1A1 by inhibiting TGF-β1-mediated phosphorylation of ERK and JNK, thereby preventing HSC activation.

To investigate whether the upregulation of NDRG2 by NPLC0393 contributes to the inhibition of HSC activation, siNC- and siNDRG2-transfected LX-2 cells were pretreated with NPLC0393 and further stimulated with TGF-β1. As expected, knockdown of NDRG2 abrogated the ability of NPLC0393 to inhibit the activation of HSCs, as evidenced by the higher expression levels of α-SMA and COL1A1 in siNDRG2 cells than in siNC cells after treatment with NPLC0393 and stimulation with TGF-β1 (Figure 5A). Consistent with this finding, the phosphorylation levels of ERK1/2 and JNK exhibited a similar pattern (Wang et al., 2010) (Figure 5B). These results suggested that NDRG2 can mediate the inhibitory effect of NPLC0393 on TGF-β1-induced activation of HSCs.

## Discussion

Liver fibrosis is a characteristic pathological feature of diverse chronic liver diseases and remains a major health problem worldwide. Despite substantial progress in the understanding of the pathological mechanisms of fibrogenesis, safe and effective therapeutic agents for liver fibrosis have yet to be explored. A previous study demonstrated that NPLC0393 can successfully attenuate experimental liver fibrosis by regulating the TGF-β1/Smad signaling pathway (Wang et al., 2010). However, this effective function of NPLC0393 cannot be performed independently, as TGF-β1 can mediate fibrosis through interacting with diverse targets (Lichtman et al., 2016; Fabregat and Caballero-Diaz, 2018). In this study, we applied TMT coupled with LC-MS/MS to screen the differentially expressed proteins in liver tissues between mice with CCl_4_-induced fibrosis and mice with CCl_4_-induced fibrosis treated with NPLC0393. The results of bioinformatic analysis and subsequent validation experiments indicated the important role of NDRG2 in mediating the antifibrotic effect of NPLC0393 and suggested that NDRG2 can inhibit HSC activation by blocking the MAPK signaling pathway (Figure 5C).

In this study performing GO biological process annotations of 413 differentially expressed proteins, we discovered that NPLC0393 can regulate many biological processes including ERK1,2 signaling pathway. Since MAPK (ERK, JNK, p38 MAPK) cascade is important mediators for non-canonical TGF-β1 pathway, we focused on proteins involved in GO term of “ERK cascade.” Among these proteins, Fn1, Lamb1 Fbln1/2, Emilin1 are important structural ECM components and provide cells with signals for adhesion, proliferation and differentiation (Schuppan et al., 2001). Integrin (Itga5) is a transmembrane receptor which can bind components of the ECM and cell adhesion molecules (Avraamides et al., 2008). TREM2 acts as a receptor for APP (amyloid-beta protein) and mediates its uptake and degradation (Yeh et al., 2016). MIF encodes lymphokine to regulate cell immunity and inflammation (Mitchell et al., 1999). ALox15, as a lipoxygenase family of proteins generate various bioactive lipid mediators to regulate inflammation and immunity (Singh and Rao, 2019). In brief, these proteins may connect different important biological processes through modulating ERK signaling. NDRG2 had been reported to participated in the dendritic cell and neuron differentiation. Since, HSC activation is also considered as a process of cell trans-differentiation (Purps et al., 2007), we were interested in evaluating the function of the NDRG2 protein in the activation of HSC.

The current study showed that NPLC0393 dose-dependently induced basal NDRG2 expression and prevented TGF-β1-induced downregulation of NDRG2. Yang et al. (2011) reported that NDRG2 exerts antifibrotic effects by inhibiting the phosphorylation of Smad3 and increasing the MMP2/TIMP2 ratio in a rat model of hepatic fibrosis. The role of NDRG2 in the TGF-β1/Smad3 pathway was further supported in a renal fibrosis model (Jin et al., 2017). In addition, a recent study demonstrated that the natural products *Salvia miltiorrhiza* and ligustrazine inhibit the proliferation of LX-2 by upregulating NDRG2 expression (Zheng et al., 2017). Therefore, we postulated that NDRG2 can mediate the antifibrotic effect of NPLC0393 in HSCs. In support of this hypothesis, knockdown of NDRG2 abrogated the ability of NPLC0393 to inhibit α-SMA and COL1A1 expression. These results indicated that NDRG2 is an important target of NPLC0393 in the inhibition of HSC activation.

Although the pivotal role of Smad2 and Smad3 in TGF-β1-mediated fibrogenesis is widely recognized, accumulating evidence suggests that non-canonical TGF-β1 signaling via MAPK (i.e., ERK1/2, JNK and p38 MAPK) activation may play important roles in certain fibrotic conditions (Zhang, 2009; Biernacka et al., 2011; Fabregat and Caballero-Diaz, 2018). Previous research by the Yang laboratory demonstrated that overexpression of NDRG2 (AdNDRG2) inhibits liver regeneration and facilitates hepatocyte apoptosis in a rat model of partial hepatectomy (PH) via Bax/Bcl-2, downstream effectors of MAPK signaling (Yang et al., 2010). Moreover, NDRG2 has been reported to inhibit the phosphorylation of ERK1/2, p38 MAPK and JNK in immune cells or myoblast cell lines to regulate the immune response and cell growth (Choi et al., 2010; Liu et al., 2010; Zhang et al., 2018). However, whether NDRG2 inhibits MAPK signaling to regulate HSC activation has not been determined. Our results showed that loss of NDRG2 triggered the phosphorylation of MAPKs and enhanced HSC activation and that these changes were prevented by treatment with ERK or JNK inhibitors. Our findings indicated that NDRG2 can inhibit HSC activation by regulating the TGF-β1/MAPK pathway.

## Conclusion

In general, via the quantitative proteomics and bioinformatics analysis combined with the literature review, we identified NDRG2 as a potential effector of NPLC0393 mediating its antifibrotic effects. NPLC0393 restored the expression of NDRG2 in TGF-β1-stimulated HSCs, and NDRG2 inhibited HSC activation by blocking the phosphorylation of downstream mediators of MAPK signaling such as ERK and JNK. Our results provide insight into the profibrotic mechanism of non-canonical TGF-β1 signaling pathway and suggest that NDRG2 could be a therapeutic target for liver fibrosis.

## Data Availability Statement

All data generated or analyzed during the present study are included in this published article or are available from the corresponding authors on reasonable request. The mass spectrometry proteomic data have been deposited to the ProteomeXchange Consortium via the PRIDE partner repository with a dataset identifier (project accession no. PXD019448).

## Ethics Statement

The animal study was reviewed and approved by Animal Care and Use Committee, Shanghai Institute of Materia Medica, Chinese Academy of Sciences.

## Author Contributions

SF, JZ, and HZ participated in the research design. HH, KW, QL, and FJ conducted the experiments. HH, KW, QL, FJ, and SF performed the data analysis. HH, KW, and SF wrote the manuscript. All the authors contributed to the article and approved the submitted version.

## Conflict of Interest

KW and FJ were employed by the company Suzhou GenHouse Pharmaceutical Co., Ltd., Suzhou, China. The remaining authors declare that the research was conducted in the absence of any commercial or financial relationships that could be construed as a potential conflict of interest.

## Footnotes

1 http://maxquant.org/)

2 http://www.uniprot.org/peptidesearch/

## Supplementary Material

Supplementary Figure Evaluation of sample and data quality.

Supplementary Table 1 Information of the identified peptides in the raw files.

Supplementary Table 2 Global protein list obtained from the mice liver tissues of the Control, CCl_4_ and CCl_4_+NPLC0393 groups.

Supplementary Table 3 Differentially expressed proteins identified from the control, CCl_4_ and CCl_4_+NPLC0393 groups.

Supplementary Table 4 GO biological process analysis of the differentially expressed proteins.

## Abbreviations

HSC: hepatic stellate cell
BDL: bile duct ligation
CCl_4_: carbon tetrachloride
ECM: extracellular matrix
FASP: filter-assisted sample preparation
GO: Gene Ontology
MAPK: mitogen-activated protein kinase
NDRG2: N-myc downstream-regulated gene 2
TMT: tandem mass tag
GOBP: Gene Ontology of biological process.
