# Supplementary material for: The Active Constituent From Gynostemma Pentaphyllum Prevents Liver Fibrosis Through Regulation of the TGF-β1/NDRG2/MAPK Axis
Source: Front Genet. 2020 Nov 4;11:594824. doi: 10.3389/fgene.2020.594824 (PMC7672159; doi:10.3389/fgene.2020.594824)
Supplement: Supplementary Figure — Evaluation of sample and data quality. [file Data_Sheet_1.DOCX]

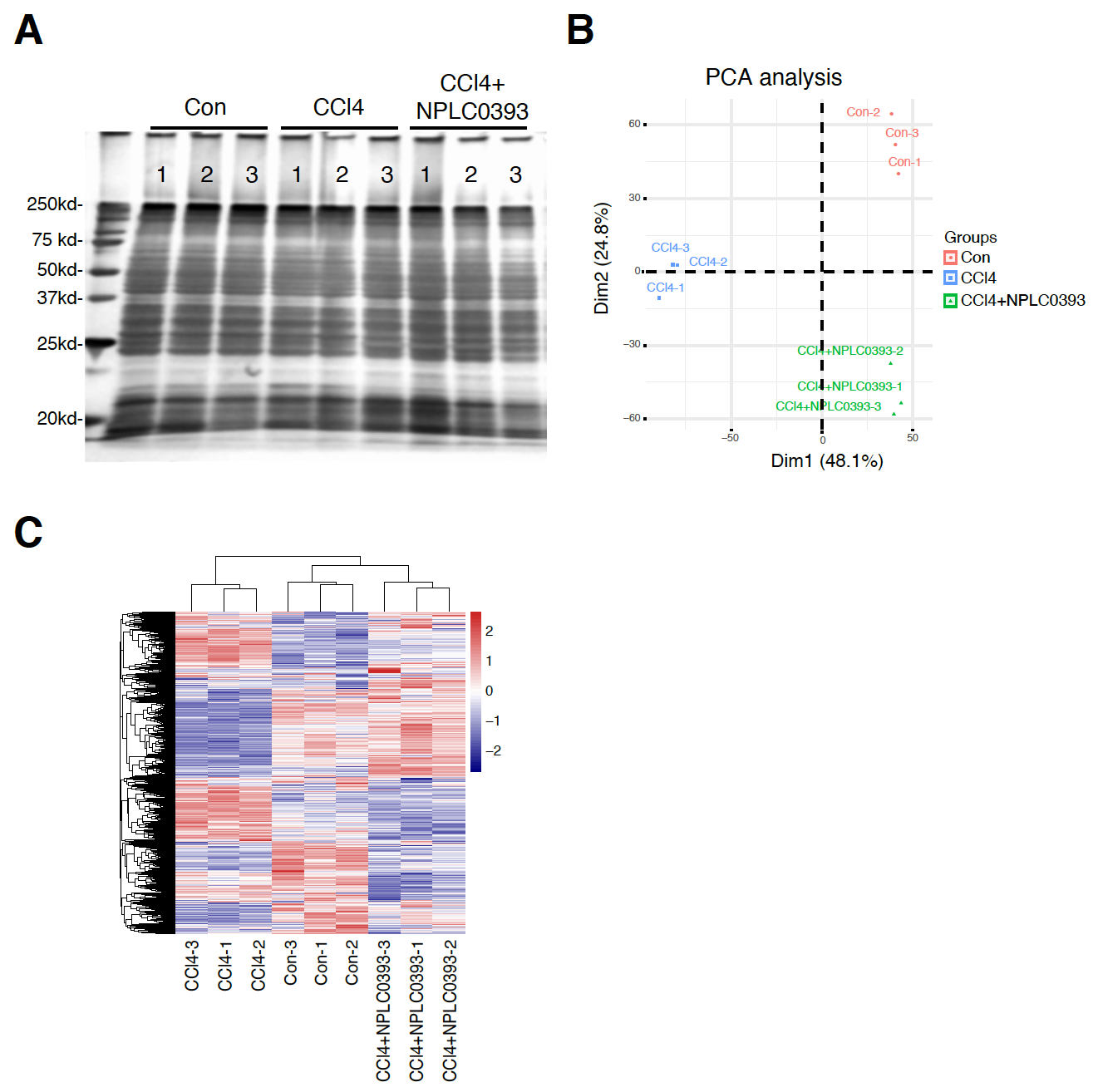


Supplementary Figure: Evaluation of sample and data quality. (A) Proteins from all samples were separated by SDS-PAGE and visualized by Coomassie Blue staining. (B) Principal component analysis (PCA) of proteomic (7344 proteins) data in 9 mouse liver samples. Red dots: control group; blue dots: CCl_4_ group; green dots: CCl_4_+NPLC0393 group. (C) Hierarchical clustering analysis of the total 7344 proteins. Columns represent different samples and rows represent proteins.
